# Supplementary material for: Odorant-Binding Proteins and Chemosensory Proteins in Spodoptera frugiperda: From Genome-Wide Identification and Developmental Stage-Related Expression Analysis to the Perception of Host Plant Odors, Sex Pheromones, and Insecticides
Source: Int J Mol Sci. 2023 Mar 15;24(6):5595. doi: 10.3390/ijms24065595 (PMC10056595; doi:10.3390/ijms24065595)
Supplement: Supplementary file 1 [file ijms-24-05595-s001.zip › ijms-2166147-supplementary.pdf]

Supplemental material

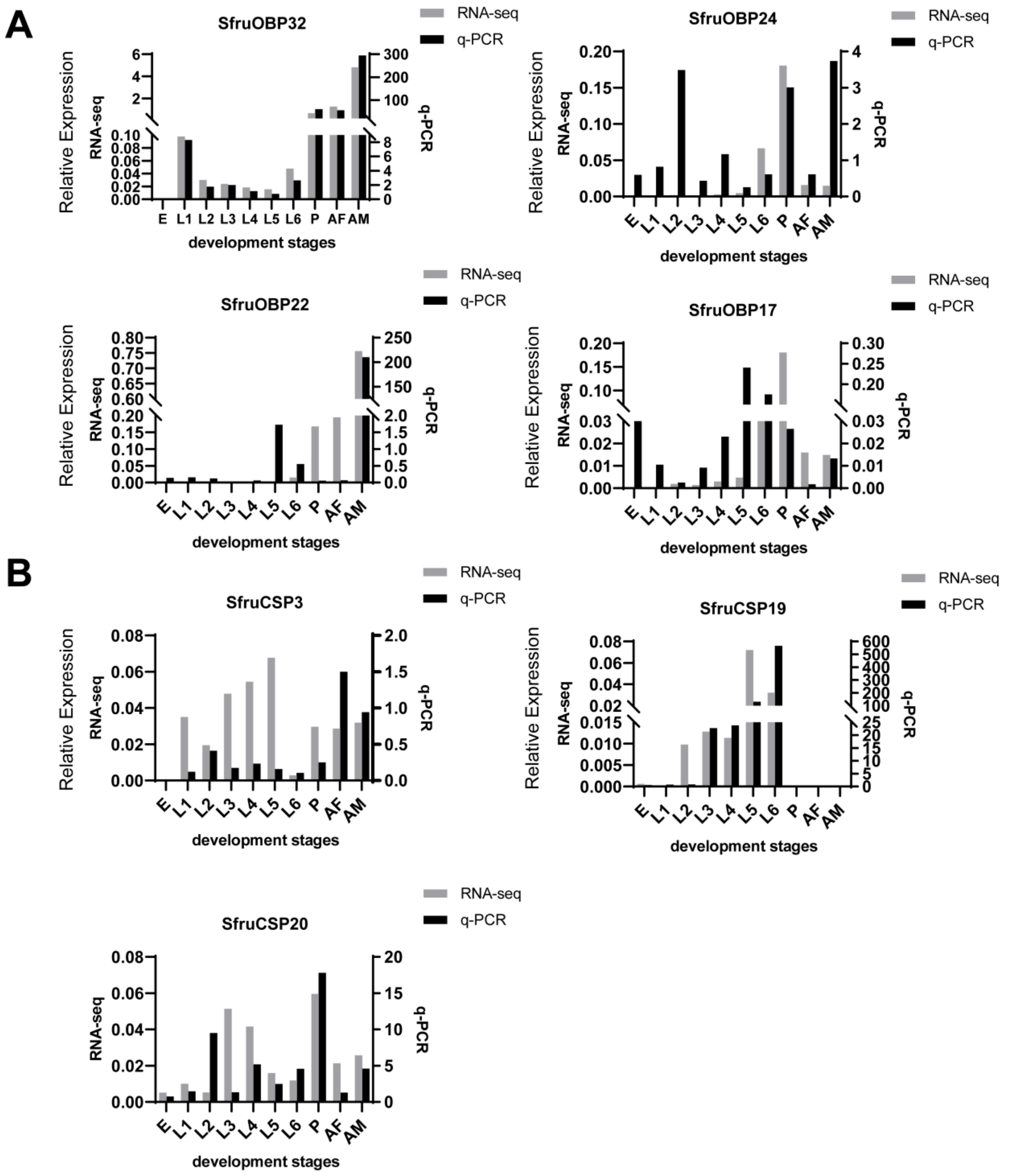

Figure S1. Measurements of relative expression levels of selected *SfruOBPs* and *SfruCSPs* by RNA-seq and q-RT-PCR. Expression level of each gene was calculated relative to reference genes of Rp132. (A) expression levels of OBPs. (B) expression levels of CSPs. Expression levels were measured across different developmental stages from eggs to adults (E: eggs; L: larvae; P: pupae; A: adult, where F: females and M: males).

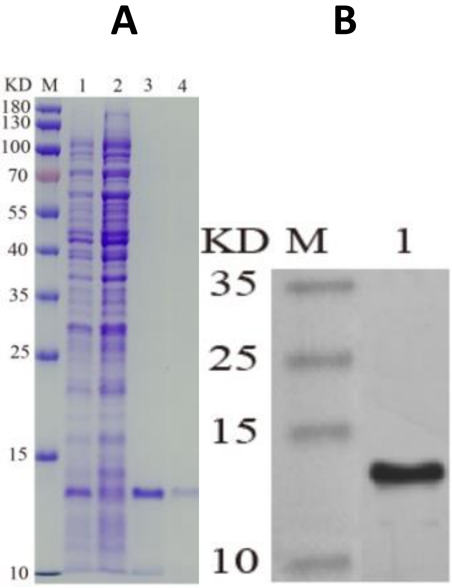

Figure S2. SfruOBP31 protein expression and purification. (A) SDS-PAGE analysis of SfruOBP31 protein. M: Protein marker. 1. Induced protein products. 2. Flow through. 3-4. Purified SfruOBP31 protein. (B) Western blot analysis of SfruOBP31 protein. M. Protein marker. 1. Purified SfruOBP31 protein.

Supplemental Table S1. Results of all *SfruOBP*s and *SfruCSP*s blastx in NCBI

Name	Description	Insect species	Max Score	Total Score	Query Cover	E Value	Per. Ident	Acc. Len	Accession	No. of conserved Cys residues
**OBPs**										
OBP1	OBP 1	*Sesamia inferens*	202	202	80%	9E-65	88.68%	106	AGS36743.1	6
OBP2	OBP 2	*Spodoptera exigua*	318	318	99%	3E-109	94.44%	162	AGH70098.1	6
OBP3	OBP 3	*Spodoptera exigua*	273	273	93%	5E-91	85.80%	170	AGH70099.1	6
OBP4	OBP 4	*Spodoptera exigua*	283	283	99%	2E-95	81.71%	164	AGH70100.1	6
OBP5	OBP 5	*Spodoptera litura*	224	224	95%	1E-72	85.50%	147	AKI87966.1	6
OBP6	OBP 6	*Spodoptera exigua*	230	620	92%	2E-69	92.00%	147	AGH70102.1	6
OBP7	OBP 7	*Spodoptera exigua*	195	473	99%	2E-58	97.92%	142	AGH70103.1	6
OBP8	OBP 8	*Spodoptera exigua*	272	272	48%	5E-89	99.31%	149	AGH70104.1	6
OBP9	OBP 9	*Spodoptera litura*	319	319	99%	5E-107	98.08%	339	ALD65883.1	5
OBP10	OBP 10	*Spodoptera exigua*	309	309	99%	6E-106	95.54%	157	AGH70106.1	5
OBP11	OBP 11	*Apocheima cinerarius*	120	120	62%	3E-30	46.62%	155	QOV03027.1	6
OBP12	OBP 12	*Corythucha ciliata*	193	193	88%	8E-60	56.85%	160	AYP30811.1	6
OBP13	OBP 13	*Corythucha ciliata*	87	87	69%	2E-17	35.81%	167	AYP30812.1	6
OBP14	SexiOBP 14	*Spodoptera exigua*	206	206	57%	7E-65	88.18%	139	AGP03460.1	6
OBP15	OBP 15	*Corythucha ciliata*	227	227	33%	3E-69	72.73%	177	AYP30814.1	7
OBP16	OBP 16	*Carposina sasakii*	268	268	98%	2E-89	78.18%	166	AYD42191.1	5
OBP17	OBP 17	*Spodoptera exigua*	258	258	94%	8E-86	91.85%	148	AKT26495.1	4
OBP18	OBP 18	*Spodoptera exigua*	317	317	99%	4E-108	79.03%	186	AKT26496.1	1
OBP19	OBP 19	*Spodoptera exigua*	224	342	54%	1E-66	65.16%	216	AKT26497.1	5
OBP20	OBP 20	*Spodoptera exigua*	228	228	68%	5E-73	78.91%	146	AKT26498.1	5
OBP21	GOBP 69a-like isoform X1	*Spodoptera frugiperda*	320	320	99%	3E-110	100.00%	154	XP_035437860.1	6

OBP22	OBP 22	*Spodoptera exigua*	289	289	99%	9E-98	94.87%	156	AKT26499.1	4
OBP23	OBP 23	*Spodoptera exigua*	479	479	99%	5E-170	94.19%	252	AKT26500.1	0
OBP24	OBP 24	*Spodoptera exigua*	324	324	99%	2E-111	100.00%	184	AKT26501.1	6
OBP25	OBP 25	*Spodoptera litura*	195	195	98%	6E-61	68.67%	154	ALD65899.1	7
OBP26	OBP 26	*Spodoptera exigua*	201	310	53%	2E-58	90.08%	149	AKT26503.1	6
OBP27	OBP 9	*Cnaphalocrocis medinalis*	281	281	75%	5E-93	77.50%	204	ALT31639.1	5
OBP28	OBP 59a	*Spodoptera frugiperda*	227	227	85%	3E-72	97.69%	244	XP_035435754.2	5
OBP29	OBP 29	*Spodoptera litura*	255	357	99%	1E-82	95.38%	156	ALD65903.1	7
OBP30	OBP 1	*Carposina sasakii*	171	171	78%	4E-52	71.30%	107	AYD42176.1	5
OBP31	OBP 31	*Plutella xylostella*	171	171	76%	7E-52	72.64%	136	ANG08529.1	6
OBP32	OBP 32	*Plutella xylostella*	216	216	99%	7E-70	78.95%	136	ANG08535.1	4
OBP33	OBP 33	*Helicoverpa armigera*	171	171	64%	3.00E-52	82.83%	99	ASA40071.1	6
**CSPs**										
CSP1	CSP 1	*Mythimna separata*	248	248	95%	1.00E-78	70.80%	290	AWT22249.1	0
CSP2	CSP 2	*Pieris rapae*	162	162	95%	2.00E-49	76.70%	100	QDW65468.1	4
CSP3	CSP 3	*Spodoptera frugiperda*	224	224	99%	3.00E-73	98.43%	127	QXT24549.1	4
CSP4	CSP 4	*Spodoptera exigua*	190	190	99%	9.00E-60	82.93%	123	AKT26481.1	4
CSP5	CSP 5	*Spodoptera exigua*	124	124	87%	2.00E-34	90.77%	131	AKT26482.1	4
CSP6	CSP 6	*Spodoptera exigua*	129	129	81%	8.00E-36	59.57%	111	AKF42439.1	4
CSP7	CSP 7	*Spodoptera exigua*	212	212	99%	3.00E-68	89.84%	128	AKT26484.1	4
CSP8	CSP 8	*Conogethes punctiferalis*	183	183	98%	5.00E-57	70.59%	120	AHX37222.1	4
CSP9	CSP 9	*Spodoptera exigua*	253	253	99%	7.00E-85	97.56%	123	AVC68635.1	4
CSP10	CSP 10	*Spodoptera exigua*	230	230	73%	6.00E-75	100.00%	122	AKT26486.1	4
CSP11	CSP 11	*Spodoptera exigua*	208	208	85%	7.00E-67	94.29%	122	AKT26487.1	4

CSP12	CSP 12	*Spodoptera exigua*	187	187	86%	2.00E-58	80.00%	125	AKT26488.1	4
CSP13	CSP 13	*Spodoptera exigua*	223	223	99%	5.00E-73	92.68%	123	AKT26489.1	4
CSP14	CSP 14	*Heortia vitessoides*	147	147	86%	1.00E-42	62.26%	128	UVB79217.1	4
CSP15	CSP 15	*Adelphocoris lineolatus*	206	206	79%	3.00E-65	79.37%	126	AXS78221.1	4
CSP16	CSP 16	*Spodoptera exigua*	234	234	80%	2.00E-76	91.80%	122	AKT26491.1	4
CSP17	CSP 17	*Spodoptera exigua*	199	199	99%	3.00E-63	93.44%	122	AVC68636.1	4
CSP18	CSP CSP18	*Lobesia botrana*	148	148	86%	3.00E-43	63.21%	127	AXF48714.1	4
CSP19	CSP 19	*Spodoptera exigua*	195	195	86%	9.00E-62	86.79%	122	AKT26493.1	4
CSP20	putative CSP 23	*Conopomorpha sinensis*	174	174	73%	1.00E-48	93.02%	557	QGN03668.1	4
CSP21	putative CSP 9	*Corcyra cephalonica*	218	218	99%	1.00E-69	62.75%	154	UDM59702.1	4
CSP22	CSP 4	*Athetis dissimilis*	239	239	85%	6.00E-78	76.87%	151	AND82446.1	4

Supplemental Table S2. Orthologues of *OBP* between *S. frugiperda* and *S. litura* that are located in the same subclades with highest similarities

S. frugiperda	*S. litura*
SfruOBP19	*SlitOBP13*
SfruOBP29	*SlitOBP30*
SfruOBP5	*SlitOBP5*
SfruOBP25	*SlitOBP25*
SfruOBP26	*SlitOBP22*
SfruOBP6	*SlitOBP6*
SfruOBP14	*SlitOBP28*
SfruOBP21	*SlitOBP26*
SfruOBP17	*SlitOBP27*
SfruOBP18	*SlitOBP18*
SfruOBP22	*SlitOBP33*
SfruOBP32	*SlitOBP12*
SfruOBP8	*SlitOBP21*
SfruOBP31	*SlitOBP3*
SfruOBP7	*SlitOBP17*
SfruOBP27	*SlitOBP31*
SfruOBP20	*SlitOBP19*
SfruOBP30	*SlitOBP20*
SfruOBP16	*SlitOBP32*
SfruOBP28	*SlitOBP1*
SfruOBP9	*SlitOBP9*
SfruOBP24	*SlitOBP10*
SfruOBP2	*SlitGOBP2*
SfruOBP15	*SlitGOBP1*

Supplemental Table S3. Orthologues of *CSP* between *S. frugiperda* and *S. litura* that are located in the same subclades with highest similarities

S. frugiperda	*S. litura*
SfruCSP21	*SlitCSP23*
SfruCSP8	*SlitCSP13*
SfruCSP6	*SlitCSP11*
SfruCSP2	*SlitCSP4*
SfruCSP20	*SlitCSP3*
SfruCSP10	*SlitCSP12*
SfruCSP16	*SlitCSP10*
SfruCSP19	*SlitCSP6*
SfruCSP4	*SlitCSP7*
SfruCSP5	*SlitCSP8*
SfruCSP17	*SlitCSP14*
SfruCSP13	*SlitCSP5*
SfruCSP12	*SlitCSP1*
SfruCSP11	*SlitCSP9*
SfruCSP9	*SlitCSP17*
SfruCSP7	*SlitCSP16*
SfruCSP14	*SlitCSP18*
SfruCSP18	*SlitCSP21*
SfruCSP3	*SlitCSP20*
SfruCSP22	*SlitCSP22*
SfruCSP15	*SlitCSP15*

Supplemental Table S4. Homologous *SfruOBP*s and *SfruCSP*s in this paper and previous report

Gene name in previous (Legeai et al., 2014)	Sf_TR2012b ID	Gene name in this paper	Annotation of inconsistent genes	identity of between two papers
OBPs				
GOBP1	c7994		Select seq ref|XP_035439875.2| partner of Y14 and mago [*Spodoptera frugiperda*] *Spodoptera frugiperda*	
GOBP2	joint2_rep_c3475	OBP2		98.77%
OBP1	rep_c2034	OBP18		100%
OBP2	joint2_rep_c9255	OBP24		99.36%
OBP3	rep_c490		Select seq ref|XP_035458708.2| GPI mannosyltransferase 3 [*Spodoptera frugiperda*] *Spodoptera frugiperda*	
OBP4	rep_c877	OBP32		99.25%
OBP5	rep_c2055	OBP10		96.18%
OBP6	c15965	OBP1		100%
OBP7	rep_c571	OBP8		100%
OBP8	joint2_rep_c7287	OBP23		99.59%
OBP9	Not found			
OBP10	c11418	OBP28		97.95%
OBP11	joint2_rep_c104	OBP6		98.4%
OBP12	rep_c504		Select seq ref|XP_035454964.2| armadillo repeat-containing protein 6 homolog isoform X2 [*Spodoptera frugiperda*] *Spodoptera frugiperda*	
OBP13	c32728	OBP19		98.65%
OBP14	joint2_c2406	OBP7		77.27%

OBP15	joint2_rep_c3798	OBP7		100%
OBP16	Not found			
OBP17	rep_c44968			97.87%
OBP18	rep_c2988		Select seq ref|XP_050560119.1| titin isoform X1 [*Spodoptera frugiperda*] *Spodoptera frugiperda*	
OBP19	Not found			
OBP20	Not found			
OBP22	joint2_rep_c1191			89.23%
OBP23	rep_c3716	OBP6		99.17%
OBP24	joint2_c2045	OBP6		91.3%
OBP25	joint2_c2317			72.8%
OBP26	rep_c2960	OBP21		100%
OBP27	c28274	OBP33		92.16%
OBP28	joint2_c6322	OBP28		99.07%
OBP29	joint2_rep_c3596	OBP29		95.65%
OBP30	rep_c4239		Select seq gb|AEA76329.1| chitin binding domain 3 protein [*Mamestra configurata*] *Mamestra configurata*	
OBP31	Not found			
PBP1	joint2_c4216	OBP4		99.39%
PBP2	rep_c1860	OBP3		99.42%
PBP3	rep_c5672	OBP12		99.39%
CSPs				
CSP1	joint2_rep_c612	CSP7		99.22%
CSP2	joint2_rep_c3099	CSP8		100%
CSP3	Not found			
CSP4	joint2_c3581	CSP22		94.38%
CSP5	rep_c3130	CSP18		94.81%
CSP6	joint2_rep_c2279	CSP9		99.19%
CSP7	joint2_c1683	CSP20		98.1%
CSP8	rep_c4613	CSP3		96.06%
CSP9	rep_c23815	CSP14		99.17%
CSP10	c10036	CSP1		77.88%
CSP11	joint2_c9626	CSP6		99.12%
CSP12	joint2_c1394	CSP4		100%

CSP13	rep_c3399	CSP17		99.18%
CSP14	joint2_c3078	CSP15		94.25%
CSP15	rep_c4149	CSP5		96%
CSP16	joint2_c2765	CSP19		96.72%
CSP17	rep_c28057	CSP10		85.16%
CSP17	rep_c475		*Gossypium herbaceum* isolate GPA1alz chromosome 5 *Gossypium herbaceum*	
CSP18	joint2_c9032	CSP16		98.55%
CSP19	rep_c601	CSP13		99.19%

Supplemental Table S5. Protein and nucleic acid sequences of *SfruOBP*s and *SfruCSP*s

Protein sequences of SfruOBPs

>SfruOBP1
MTLIKKKSKFGEIVKRTVIATAHTCMDHVNATTKDLEHLRDEPPYPETSACIVKCLLEKIGVVKS
NRYSKMGFMTAVTPLVFANKKKMEHMKTVSENCDKEVNHKHETPCQLGNEVTTCIFKYAPELHFK
S
>SfruOBP2
MTSKCCLLLVVMATVTSSVMATAEVMSHVTAHFGKALEECREESGLSAEVLEEFQHFWREDFEVV
HRELGCAIICMSNKFSLLQDDSRMHHVNMHDYVKSFPNGHVLSEKLVELIHNCEKKFDSMTDDCE
RVVKVAACFKVDAKAAGIAPEVAMIEAVMEKY
>SfruOBP3
MTLRQSGTMSVRVALVVALVVAASLLVVQASQDVMKNLAINFAKPLDDCRKEMDLPDSVTTDFYN
FWKEGYELTNRQTGCAILCLSSKLEILDQELNLHHGRAQEFAMKHGADEAMAKQIVDMIHTCAQS
TPDVAADPCMKTLNVAKCFKDKIHELNWAPSMELVVGEVLAEV
>SfruOBP4
MADARWRVVCVVCALCLTSAVLASQDLMVKMTKGFSKVVDDCKTELNIGDHIMQDMYNYWREEYQ
LINRDMGCMVLCMAKKLDLMEDQKMHHGKAEEFAKSHGADDDVAKKLVSIIHECEQQHAGVADEC
MRALEVAKCFRTKIHELKWAPSMEVIMEEMMTAV
>SfruOBP5
MFVVRCSSFVVALFCLVSVYAQSDEEAGIKEALRPFVEECADEFGITEEQFEEAKKKGNAADIDP
CFMSCFLKKAGFFDSQGKFDVDSTMAFAKDHVSSEPAMKFVEAVGDECVKINDEDVSDGDKGCDR
AKLLFDCIGETKKKME
>SfruOBP6
MSKFACLVLCVVAVSLSGVHATAEEKAAFIEAVKPHIQECSKEHGVTPEEIKSAKAAGNADGINS
CFLSCVYKKAEVINDKGEYDADKALEKLKKFVSNEDDYAKFAEIGKKCASVNEKSVSDGDAGCER
AALLTTCFLEHKSEIPA
>SfruOBP7
MTRQQLKNSGKLMKKSCMPKNDVTEDEVGDIEKGKFIESRNVMCYIACVYTMTQVVKNNKLSYEA
VIKQVDVMFPAEMRDAVKAAATHCKETTKKYKDLCESSYWTAKCMYDYDAENFVFP
>SfruOBP8
MARRQRGAMFTETLPLFVILVAVTHGGKDKPVFSDEIKEIIQTVHDECVAKTGVAEEDITNCENG
IFKEDAKLKCYMFCLLEEASLVDDDDTVDYDMLVSLIPDEYYERTTKMIFACKHLDTPDKDRCQR
AFEVHKCSYEKDPDLYFLF
>SfruOBP9
MGQYNNNYYSTTQSSRRYKRERRPSNSGQRSQYNPNSHKISGYEDNFRSDERNNTENNSSKETDN
KSCALHCFLENLEMTGEDGMPDRYLVTHAITKDVKNEDLRDFLQESIEECFQILDNENTEDKCEF
SKNLLICLSEKGRANCDDWKDDLTF
>SfruOBP10
MTKVLFAIVLTMITFAVALSASTKEAQTTTMSDQVNSIEVDVLAVMDMCNDSYRIDPTYLQALNE
SGSFIDETDKTPKCFIRCVFENVGIVSEDGKQFNPARAAVIFAGERNGKPMEDIADMTALCATDR
QETCPCDRSYKFLRCLMSMEIERYEKS
>SfruOBP11
MCKFRVLLLGFAVVAVYFSHVNCLYDEDKKILRDGLESIAHDCLHGCGIDETEIENIEADDNVDQ

CFKKCVMTDAGFALSDREKSTIQKELTTVGLQCIQQHPLSLSDIRSFRNKMIPDGKKPKCFVACL
FKKIGVMDDMGMISPMKAEENAKKVFKDNEEHIKHVNEIMEKCSSVNHQNTSDGNKGCDRAKLAF
NCFTENADRYGFDFDF
>SfruOBP12
MGSCNVFVALVVLTVGVREIEPSKDAMKYITSGFVKVLEECKQELNMNDHIIADLFHYWKLEYTL
LNRDTGCAIICMSKKLELLDANGRMHHGNAQEFAKKHGAGDEVASKIVQIIHECEKKHERDDDEC
LRVLEVAKCFRTGIHELDWQPKVEVIVEEVLTEM
>SfruOBP13
MLGMKTIVVLCLIVYEAGLVHGEHEVSVLRALSTSIGDTLLECQTELDIKNEVIQSFLNFWDLKN
PADTKEWGCALDCVFQKNAFLTPDGKTVISANVREFCKAAGADELMSVRMVDLFEMCKDGAKKII
SKCDNALEVTKCFRHGIVQLDWAPEHNFWNRTREHPDNIPVQRTDIVKIPLPQRHSSLNVWRRRF
GYRRLASILRRSFC
>SfruOBP14
MMRKTCQPKNNVEDEKIDPIADGVFIDEKEVKCYMACIMKMANTIKNGKLNYDAAIKQADLLLPD
DIKEPAKEAITACRKVGMDCNIEHPITPKEMLELKDNKIPDTNNAKCFVACVFKKTGMLDSKGMY
DAENSIAMTQKDFANDPKRLEGSKKLLETCKKVNDEAVSDGEKGCERSVLLHKCFVETAPQ
>SfruOBP15
MLLLLRALPLLAAVLPLRADVNVMKDVTLGFGQALDKCRQESQLTEEKMEEFFHFWRDDFKFEHR
ELGCAIQCMSRHFNLLTDSSRMHHENTEQFIQSFPNGEVLARHMVELIHACEKQHDHEEDHCWRI
LHVAECFKQACVQRGVAPSMEMMMTEFIMEAEAR
>SfruOBP16
MEKILIFTTFLSLAVVVNSFKTKFFSQSLDSEPPVSILYARDKKSDKVTDDCLTEMYPRNLYKYP
LRIDRNDIPCIIHCVLKKFGIMTNDGYINIKNYYRRVQAIHRYDPRILISDVGETCAQNINGMNL
DHDVCKKAKVFNDCTQLYAVSYKDPDEWK
>SfruOBP17
MAQHCNNASHADVVQTPTKRETRASLKPLSVCCDIPELADERQLSKCSPKPPGPCEDVQCIFEAS
GFLTDKNTLNKAAYRSHLQKWEKSNPGWTDAIYKAITDCVDNDPRQHLDVACKAYDVFTCTGIAM
LKKCPETAWKC
>SfruOBP18
MFKFCVFLALGFVACHGAPNTNPGTYCGATPDNMFRCLNNPRVVTPEVAAKCGSQVTECERMTCI
FRELKWSKKGAIDKAKVRAYFEQYETEHPDWAQAVQHVKSFCLVPELRAQGVFLNCPAYDIMQCI
LSSFIKHASPSVWSNATDCDYPKAFAAACPVCPSDCYSPQIPFGSCNACYSQPRTV
>SfruOBP19
MITSSSLLVLAAVVQVLFAQEPAFESGPPEPWGPPEKQTPPRLAPRIPRNCWAPPQRINVYKCCP
IPSLYPDEAMQSCGFEKLPEGEAPKKAVYRPEGTCKEGYCVMEKFNLLLANKSVDYEKFGNYLDS
WAEANPEFAKPIKLAKEECAKDEGPKGPPICDPDRIFLCLTSIIFWNCDLRDGEGCSALQEHMNE
CRQYYTR
>SfruOBP20
MFRRALLFLSVIYSLQYCNGQTEAPEKNRMMGIDAVHDNNVKIDKDTIITRNLKLEKRNRGPKSD
SNKNVDEKEPDWSYASFPKEISEHVENFKKNMSECLKEVQSSDKRPVKRLSPKMESPVHGECLIA
CVLKRNGVIIHGKVNKDNLIALVSKFYSKDTRLMKKLEKNLDRCIEMSVRAQDDCTLASLLNDCT
NDLMASNKHKLTVNY
>SfruOBP21
MFKLSVFVCCLYFCALTPYLVSAMTAEQKAIIHEHFETIGKACNKDSTVITADDVVNLRARKMPS

GPNAPCFLACMMKQIGIMDDNGMVQKENVLEMAKAVFDDPEELKAIEDYLHSCSQVNTESVSDGA
AGCERAMLAYKCMIENASKFGFDI

>SfruOBP22

MTCSQALALLALVAISQQATTGCKNCIMLGKEEKAMFRAHSDACVAASRVEPRLVDAMLAGELLD
EPALRRHVYCVLLKCKLISKDGKLQKAAVLGKMAARPDAKNATKVLESCADQTGDTPEDLAWNLF
RCGYDKKALLFDYMPTNVASETDNNS

>SfruOBP23

MILSVCYGAVDIKKYFKVCDRNAIDVNDCMAEAVRQGIATMINGIDELGVPPIDPYLQKEFRMEY
KNNQIAAKLNLKNIYVEGLKEAIVHDARLRADDDKFHLEVDLSGPRVSVKADYYGEGQFNSLKIV
AYGQVNTTMTDLVYTWKLDGVPEKNGTETYIRITDFYMRPDLGSIVTLFKNDNPESRELTDLGTR
FANENWRTLYKEFLPYAQANWKRIGVRVANKLFLKVPYDQLFPTSS

>SfruOBP24

ESRCRNPPTAPQKIERVITLCQDEIKLSILREALDVIKEEHTMPAQRRRDKREVPFTHDEKRIAG
CLLQCVYRKVKAVDGYGFPTLEGLVGLYSDGVNERGYFMAVLEASRECLMKNHDKFSRTVPMDNG
RNCDVSFDIFECISDRIGEYCGTSGL

>SfruOBP25

MAKVTCILLFVVAVSLSSVQTDESQNSSEVDADVDKVIDECAKEFHVPHQLLMAAVTTGSVHALT
PCFWSCCFKGVGVLNSEGQYDIDATLDLSKKMFSGSEYQKVEGIVKSCGSVNDISVSDGKAGCEI
SVSLAVCILDNCKKIFPNMFHD

>SfruOBP26

GCERAVLGAICYMEHKTVQAAVSEEEKEAFREAMAPIIAECSEEHGVSEADIKAAKEAASADGIK
PCFLGCVMKKIEVLDSKGLYDAETGLGKLKKFVKDEADFAKFEEIAKKCLKVNEESVSDGEEGCE
RAKLILGCFIDHKVEMPF

>SfruOBP27

MKYKTRRTRNEREQEKMHKLQNIFALILFILFSFAFYLTISFTPLTKEEQMERYNKMTENVEPFR
KNLTECARQVKASMADVENFIKRIPQASLQGKCFVACILKRNSIIKNNKISKEHLLEANKAVYGE
DSEVMARLKTAVGECTQVVEGIFEVCEYASVFNDCMHIKMEHILDKVTMERRMEAIGKMTSNPDQ
WNEEEDELLKLVKDEL

>SfruOBP28

MVGAVALLALLPALVAGAGEGNIKLLEDEVAVALKSCTYPDDITNSKEPASKERQRRSEDSYDAS
PRIDDNVKEGNRYSHERRISNDSGDQLMVINATDDDYSGYGSGNMGEKLLTSVPRPASPNNNINK
NNTSRTRRNEPLLNRPDSDQCLSQCVFANLQVVDSRGIPREAELWNKVQSAVTSQQSRSALHDQI
RACFQELQSEAEDNGCSYSNKLERCLMLRFADRKVDGKGNAKKSSKEQT

>SfruOBP29

MWNSLFFLFTFGVFSLNAEALTMDDLKQKYVDNILECSKQYPVDRADAEQLQNRIMPDKESVRCL
FACVYKLAGMMNDQGELSVEGVNAISRKYLAEDPEKLKKSEQFTEACRSVNDAPVNDGTRGCDRA
ALIFQCTIDKSPDGLTEEELKMEFTKLVMKCNKDGQMDMTELVQLQNYVVPTKQSAKCVLACAYK
AAEVMNAKGEYDIDHAYKVAEMMKNGDEKRLVNAKKMADLCVKVNENSVSDGEKGCDRAAMIFKC
TVENAPKFGFKL

>SfruOBP30

MVDKILIFTFITLSGFAHARMSVMYAHDKLSDIVAQQCLSEMYPKNRRIEIQESDEPCIIFCVLK
KFGIISATGVINLEVYRKRVLIAHQLDQNTSVMDNGGSCMENAEATQHKQDVCKKAKVFNDCTHL
YRILLM

>SfruOBP31

MWTQALVLTLGALASLAAAAVEMDEDMAELARMVRDNCAGETGVDVALVEQVNAGADLMPDEKLK
CYIKCTMETAGMMADGEVDIEAVLALLPPSLADQHAPSLRACGTQRGADHCDTAFRTQQCWQKAN
KADYFLI
>SfruOBP32
MKTLLVFAACILLAHALTDEQKEKLKKHRSECLTETNVDEQLVNKLKGGDYKTESEPLKKYALCM
MMKSELMTKDGKFKKDVALAKVPNPADKPTVEKLIDACLANKGNTPHQTAWNYVKCYHEKDPKHA
IFL
>SfruOBP33
MFKSGVVYCIVVAVFFKNALAITDELKSHIEAKFLTVGAECIKEHPLTIEDLSAFKNRVFPEGEN
AGCFSACIFNKLGLFDDKGSLSHLTALENAKKVFEDQGELENIEKFLTTCAKVNDEEVSDGEKGC
ERAKLAYNCFIENYEQLGFDLEF

Protein sequences of SfruCSPs

>SfruCSP1
MVRLKVKIGILPEALRTKCVRCTERQKRTAVKVIKRLKNEYPDEWSKLASRWDPTGDFTRYFEEF
LAKEHYNTIPGSGSALPTSSPLAPPRVPQSVPATTPTPGPTESTPPRPIVLNRFGGDGELMMGSP
SSAGVTPRPMTQATTRPSTTTKPSTTTKSPSTRPVPPRPTMMTWAGAASNTQSTRFPLRPVSEMS
PPYSTAITFIDQIGYKIIRTTELVTDIVRNTVRAVVGR
>SfruCSP2
MQIILVLIVACVGLAAGLHVQAGPQMTDAQLEQTLADKSTMQRHIKCALGEGPCDPVGRRLRTLA
PLVLRGACPQCSMQETRQIRRTLAFVQRNYPWEWAKIVRQYG
>SfruCSP3
MKSMLVLCVLSVAALVVARPEDHYTDRYDNVNLDEILSNRRLLVPYVKCTLDQGKCAPDAKELKE
HIREALENECGKCTETQKKGTRRVIEYLINNEEAYWNELTAKYDPERKYTVKYESELKKIKA
>SfruCSP4
MKCIYVLSALLVFAAVQAEDKYSTENDDLDIDAVVADVDALRGFVGCFMDSVTCHAVAADFKKDL
PEAVATSCSKCTEAQKHIFHRFLLGLKQKLPADYEAFKKKFDPEGQHFHALEANVSNS
>SfruCSP5
MNGANKYNFGLLLIFMCLFIATVVSQEKFYDRRYDYYEIDTLIQNPRLLKKYLDCFLWRGPCTPI
GRVFRQILPEAVQTACKKCTQSQRRLARKTFDAFKGYFPEIHEELRKRLDPKNKYYEAFEKAISS
A
>SfruCSP6
MKVLVVLSVFLALAAAAPPKPITKEELAILEAFDYDAVFANEESRKLIFDCLLDKGDCGPYKKVV
ELSKKSMYDECALCSPTQMAKYDRVLKLLHDDYETFYNELIKKVASEKE
>SfruCSP7
MKFVLVLCLVAAAVLADDDKYTSKYDNIDLDEILSNKRLLTAYVNCVMERGKCSPEGKELKEHLV
DAIETGCKKCTEAQEKGAYKVIEHLIQHELDTWRELCDKYDASGKWRKTYEDRAKANGIVIPE
>SfruCSP8
MKVALLTLCFALGVLAQDMYENANDNFDISEVLGNERLLNSYAKCLLNKGPCTPEVKQVKDKLPE
ALETRCAKCTDKQKQMGKTLAQEVKKNHPDIWKQLVEMYDPEGKYQQAWKDFLQE
>SfruCSP9
MNFLVLSIVVTMAAFVAAETYTDRYDHINIDEIIENRKLLVPYIKCTLDQGRCTPEGRELKAHIK
DAMQTSCSKCTEKQKKGARKVVRHIRAKEQDYWKQILAKYDPEDQYKENYESFLAAED
>SfruCSP10

MGPQPSKPGIATVDAARGALAQLSFGKYFSIKSFLESAVKVFINMRSWLLCLCVLTVVVSCYSQA
NRYENFNPDAIVQNDRILLAYYKCVMDKGPCTRDGKNFKRVLPETLATACGRCNPKQKTIVRKLL
LGIRSKSEPRFLELLDKYNPDRSNRDALYAFLVTGA
>SfruCSP11
MRVLVALSCLVVVALAADKYNAKYDNFDVDTLISNDRLLKAYINCFLDKGRCTPEGSDFK
KALPEAIETTCAKCTEKQKGNIRKVIKAIQQKHPKEWEDLVKKNDPSEKHRANFDKFLQG
SS
>SfruCSP12
MKLVIILALVAVAVARPDDGGTYDSKYDNFNVDELVGNARLLKSYAHCFLDDGKCTPEGNDFKKW
IPEATTTSCGKCSEKQKALVAKTIQAIKDKLPAEYEALIKKHDPENKHQGKLQEFLQKYSH
>SfruCSP13
MKSILVLCLLVAAVSCKPETYDTRYDNFDVESLVGNVRLLTAYGHCFLGNGPCTPEGSDFKKTIP
DALRTGCGKCSPKQRHLIRVVVAGFQSKTPDLWQQLVKKEDPNGQYKEIFTRFLNARD
>SfruCSP14
MKVVFLVCVLAAVVYGEQYTDKYDNIDLDEILHNEKILQSYVNCCLDQGKCTPDGKELKSHIKEA
LENRCAKCTDAQKRGTEKVLKFLINNKPDIWDQLCNKFDPEGLYRKKYEQEYQSAKQ
>SfruCSP15
MQYIFRYGRDEGVTAQRRVERSSREIRYKMKLIIVLALCVVGVAWARPASTYTDKWDNIN
VDEILESQRLLKGYVDCLLERGRCTPDGKALKETLPDALEHECSKCTEKQKKSSDKVIRF
LVNKRPDLWQELSGKYDPENIYQERYKTQLEAAKKH
>SfruCSP16
MYGIKLDEWWLQPSIPLGHRIIHIDTKFNMNALLIAVFALAAPLAYGYDEKYDKIDVDKI
LGDDALFTTYINCMLDKGPCSVEHSADFRQLLPEVIATACEKCNAIQRQNVRKTVKALSE
KKPDEFAQFRAKFDPKGEYEKAFSAFVIGTD
>SfruCSP17
MKADCVLIMTLMAVVAADFYNSKYDNFDVQPLLENDRILLSYTKCFLDQGPCTPDAKDFKKIIPE
ALETTCGKCSPKQKQLIRTVIKAVIAKHPEAWEQLSDKYDKDKKYKDSFDKFLAEQE
>SfruCSP18
MGNYVEIIVLELKEDKLLTKALHREIMGLMDDSEVCNDTLEPHYMYTVFTMRSAVVLWNMIVEVT
LTVDSAVRQKMKSFIVLCLFGLAAVALAKPNGSTYTDRYDNVNLDEILGNRRLLTPYIKCILEEG
KCTADGKELKSHIREALEQNCAKCTDAQRSGTRRVLGHLINNEEESWNRLKAKYDPQSKYTVKYE
LELKKLKQ
>SfruCSP19
MKFVLVVFVAAFAVVMAEESYGTQYDNVNGEAIVSDDKQFQSIVDCFMGADSCNELAAAFKKVLP
EAIVQACAKCTSAQKHLARVFIEAYSKKMPQDYEKFKDLFDPERKYFAKFEASVTGF
>SfruCSP20
MGNCCSKLPTMIKYALLLCCVAAVSVAQSQRPPVSDTALDDALQDKRFIQRQLKCALGEG
PCDPIGKRLKTLAPLVLRGACPQCTPQETKQIQRTLSYVQRNYPQQWAKIVRQYAG
>SfruCSP21
MWAQAIPYLIVVHVAMGEQKVPGFSREMSDGVESRGFKIIYGDEDMTIINQVVSEAEKGSAFKKR
VRLNEAIKPLPAQDVKCLMSVDRYCSKEMGMMKSVLIQAVKDDCAKCSVEQKDQAGKVIASMMAH
DPVAWKVFLTRYDGIKKVQRILG
>SfruCSP22
MASSYYSVLTVNETVISSAVPGMMKNSYKTMILLVYLTIQSNATETSTYTTKYDGIDLDEILDND

RLLTGYVNCLMDLGPCTADGKELKKNLPDAIDNDCKKCTERQREGADRVCHYLIDNRPEDWTKLE
EKYKSDGSYRAKYLASKQTKDEKESNATKSSEDTNNVSKE

Nucleic acid sequences of SfruOBPs

>SfruOBP1
ATGACCCTCATAAAGAAAAAAAGCAAATTCGGCGAGATAGTGAAGAGAACAGTCATCGCCACTGC
ACACACATGTATGGACCATGTCAACGCCACAACTAAAGACCTGGAGCACCTTCGTGATGAACCGC
CCTACCCAGAAACTTCAGCTTGCATTGTTAAATGCTTACTAGAAAAGATCGGTGTTGTGAAAAGC
AACAGATATTCGAAAATGGGTTTCATGACCGCCGTAACACCTCTAGTGTTCGCAAACAAAAAGAA
AATGGAACACATGAAAACAGTGTCAGAAAACTGCGACAAAGAGGTAAATCACAAACATGAGACGC
CATGTCAACTTGGAAATGAGGTCACTACATGCATCTTTAAATACGCTCCCGAGCTACATTTTAAG
TCTTAA
>SfruOBP2
ATGACGTCGAAGTGTTGTTTGCTGCTGGTCGTCATGGCAACCGTCACCAGCTCGGTGATGGCCAC
CGCCGAGGTGATGAGCCACGTCACCGCACACTTCGGGAAGGCTCTCGAGGAGTGCAGAGAAGAGT
CAGGGTTATCAGCAGAGGTGCTGGAGGAGTTCCAGCACTTCTGGCGCGAGGACTTCGAGGTGGTG
CACCGCGAGCTGGGCTGCGCCATCATCTGCATGTCCAACAAGTTCTCGCTGCTGCAGGACGACAG
CAGGATGCACCACGTCAACATGCACGACTACGTCAAGAGCTTCCCTAATGGCCACGTGCTATCGG
AGAAGCTGGTAGAACTGATCCACAACTGCGAGAAGAAGTTCGACAGCATGACGGACGACTGCGAG
CGCGTGGTGAAGGTGGCGGCCTGCTTCAAGGTGGACGCGAAGGCCGCCGGCATCGCGCCCGAGGT
GGCCATGATCGAGGCCGTCATGGAGAAGTACTGA
>SfruOBP3
ATGACGCTCCGGCAGTCGGGTACCATGTCGGTGAGGGTGGCGCTGGTGGTGGCGCTGGTGGTGGC
CGCCAGTCTGCTGGTGGTGCAAGCCTCGCAAGATGTCATGAAGAACTTGGCCATCAATTTCGCGA
AACCATTGGATGACTGTAGGAAGGAGATGGACCTGCCGGACTCGGTGACGACAGACTTCTACAAC
TTCTGGAAGGAAGGCTACGAGCTGACGAACAGACAGACCGGCTGCGCTATCCTCTGTCTCTCCTC
CAAGTTGGAGATTCTCGACCAGGAACTGAACCTACACCACGGCAGGGCACAAGAATTTGCCATGA
AACACGGAGCTGACGAGGCCATGGCGAAGCAGATAGTGGACATGATCCACACGTGTGCGCAGTCC
ACCCCCGACGTGGCGGCCGACCCTTGCATGAAGACCCTGAATGTAGCCAAGTGCTTCAAGGACAA
GATCCACGAGCTCAACTGGGCGCCCAGCATGGAACTCGTCGTGGGAGAAGTACTGGCTGAAGTGT
AA
>SfruOBP4
ATGGCGGACGCTAGATGGAGGGTTGTGTGTGTTGTGTGCGCGCTGTGCCTGACCAGCGCCGTGCT
CGCCTCGCAGGACCTCATGGTCAAGATGACCAAGGGATTCTCGAAGGTCGTGGACGACTGCAAGA
CTGAGCTGAACATAGGCGACCACATAATGCAAGACATGTACAACTACTGGCGCGAGGAGTACCAG
CTCATCAACCGGGACATGGGCTGCATGGTGCTGTGCATGGCCAAGAAACTGGACCTCATGGAAGA
CCAGAAGATGCATCACGGGAAGGCAGAAGAATTCGCCAAGAGTCATGGAGCTGACGACGACGTGG
CCAAGAAGTTGGTGAGTATAATCCACGAGTGCGAGCAGCAGCACGCCGGCGTGGCGGACGAGTGC
ATGCGCGCGCTGGAGGTGGCCAAGTGCTTCCGCACCAAGATCCACGAGCTCAAGTGGGCGCCCAG
CATGGAGGTCATCATGGAAGAGATGATGACCGCCGTATAA
>SfruOBP5
ATGTTCGTTGTGAGATGTTCCTCTTTTGTGGTGGCGCTGTTTTGTTTAGTGAGCGTTTATGCTCA
ATCAGATGAAGAGGCAGGTATAAAAGAGGCTTTGCGCCCTTTTGTAGAAGAATGTGCAGATGAAT
TCGGTATTACTGAGGAACAATTCGAAGAAGCAAAGAAAAAGGGCAATGCTGCTGATATCGATCCT

TGCTTCATGAGCTGTTTCTTAAAGAAAGCTGGATTTTTTGACAGCCAGGGTAAATTTGACGTAGA
CAGCACCATGGCTTTCGCCAAGGACCATGTGAGCAGTGAACCGGCTATGAAGTTCGTTGAAGCTG
TCGGAGATGAATGTGTTAAAATTAATGATGAAGATGTCAGTGATGGAGACAAAGGATGCGATAGA
GCTAAACTGCTGTTTGACTGTATAGGTGAGACTAAGAAAAAGATGGAGTGA

>SfruOBP6

ATGTCTAAGTTCGCTTGTTTGGTTTTGTGTGTTGTGGCTGTGAGCCTGAGTGGGGTTCATGCAAC
TGCCGAGGAGAAAGCAGCTTTCATAGAAGCTGTGAAGCCCCATATACAGGAGTGCTCAAAGGAGC
ATGGAGTCACTCCTGAAGAAATTAAATCTGCCAAGGCTGCAGGCAATGCAGATGGCATCAACTCT
TGTTTCCTGAGCTGTGTTTATAAGAAAGCTGAAGTTATTAATGACAAAGGAGAATACGATGCCGA
TAAAGCCCTGGAGAAACTCAAGAAATTCGTGAGCAACGAAGATGATTATGCTAAATTCGCAGAAA
TTGGAAAGAAATGCGCTTCAGTTAACGAGAAGTCAGTAAGCGACGGAGATGCTGGTTGCGAAAGA
GCTGCACTGTTGACCACATGCTTTTTGGAACACAAGAGCGAGATCCCCGCTTAA

>SfruOBP7

ATGACACGACAACAGCTAAAAAATTCTGGCAAACTTATGAAAAAATCCTGCATGCCGAAGAATGA
TGTGACCGAAGACGAAGTAGGTGACATCGAAAAGGGGAAGTTTATAGAAAGTCGTAACGTTATGT
GTTACATCGCATGTGTCTACACAATGACTCAAGTTGTGAAGAATAACAAACTAAGCTACGAAGCT
GTTATTAAACAGGTGGATGTGATGTTTCCAGCTGAGATGAGGGATGCTGTCAAAGCAGCTGCTAC
TCACTGTAAAGAAACAACCAAGAAATATAAAGACTTGTGTGAATCATCATACTGGACCGCTAAAT
GCATGTACGACTACGACGCAGAAAATTTTGTATTTCCTTGA

>SfruOBP8

ATGGCGCGGAGGCAGCGGGGCGCCATGTTCACAGAGACGCTGCCCCTCTTTGTCATTTTAGTTGC
TGTTACTCATGGCGGTAAGGATAAGCCGGTGTTTAGTGACGAAATCAAAGAAATAATACAAACAG
TTCACGACGAGTGTGTCGCTAAAACTGGGGTCGCGGAGGAGGATATAACGAATTGCGAAAACGGC
ATATTTAAGGAGGACGCGAAATTGAAGTGCTATATGTTTTGCCTGCTGGAGGAAGCGAGTCTCGT
CGATGATGACGATACAGTGGATTACGACATGCTCGTCAGTCTGATACCTGACGAGTACTACGAGA
GAACTACTAAAATGATATTCGCATGTAAACATCTCGATACTCCAGATAAAGACAGATGCCAGAGA
GCGTTCGAAGTACACAAATGTTCATATGAAAAGGATCCAGACTTATACTTCTTATTCTGA

>SfruOBP9

ATGGGTCAGTATAACAACAACTACTACTCCACAACACAGTCCTCCAGGAGATATAAAAGGGAGCG
ACGTCCGAGTAACTCGGGACAAAGGAGTCAGTACAACCCGAACAGCCACAAAATCTCAGGATATG
AAGATAATTTCCGATCAGACGAGAGAAACAATACAGAAAATAATTCCAGCAAAGAAACAGACAAT
AAGTCGTGTGCGTTACACTGTTTCTTGGAAAATTTGGAGATGACAGGGGAGGACGGCATGCCGGA
CAGATATCTGGTGACTCATGCCATCACGAAAGATGTGAAGAATGAGGACCTGAGGGATTTCCTCC
AGGAGTCCATTGAAGAGTGCTTCCAGATTCTCGATAACGAAAATACCGAAGACAAATGCGAATTT
TCGAAAAACTTGCTGATTTGTTTGTCAGAGAAGGGAAGGGCTAACTGTGACGATTGGAAGGATGA
CTTAACGTTCTAG

>SfruOBP10

ATGACTAAAGTACTATTTGCCATCGTAGTGACGATGATTACATTTGCCGTAGCACTCTCCGCCAG
TACTAAGGAAGCTCAGACCACCACCATGTCTGACCAGGTTAACAGCATCGAGGTAGATGTCTTAG
CTGTTATGGACATGTGCAACGATAGCTACAGGATTGATCCAACTTATTTACAAGCACTAAATGAG
AGTGGGAGCTTTATTGATGAAACTGACAAAACGCCAAAGTGTTTCATCCGCTGTGTTTTCGAAAA
CGTTGGCATAGTATCTGAGGATGGTAAGCAGTTCAATCCTGCGAGGGCAGCTGTCATCTTTGCTG
GGGAACGGAATGGAAAGCCTATGGAAGACATCGCAGACATGACTGCTCTGTGCGCCACTGATAGA
CAGGAAACCTGCCCCTGCGACAGATCATACAAGTTCCTAAGATGCCTCATGTCAATGGAAATTGA

AAGATATGAGAAATCTTAG
>SfruOBP11
ATGTGCAAGTTCAGAGTGCTCTTGCTGGGCTTTGCAGTTGTGGCGGTATATTTTAGTCACGTTAA
TTGCTTATATGACGAAGACAAGAAAATTCTACGTGATGGTTTGGAGTCTATAGCTCATGACTGTT
TGCATGGATGTGGTATCGACGAAACAGAAATAGAAAATATTGAAGCCGACGATAATGTTGACCAG
TGTTTTAAGAAATGCGTTATGACAGACGCTGGATTTGCATTATCAGATAGAGAAAAGTCAACTAT
TCAAAAAGAGTTAACAACAGTTGGTCTACAATGTATACAGCAACATCCTCTATCTTTGTCTGATA
TTAGGTCGTTTAGGAACAAAATGATCCCTGATGGTAAGAAACCAAAGTGTTTCGTGGCCTGCCTG
TTCAAGAAAATTGGAGTGATGGATGACATGGGAATGATATCACCAATGAAGGCAGAGGAGAATGC
TAAAAAGGTCTTCAAAGACAACGAGGAGCATATTAAACATGTCAATGAGATTATGGAGAAATGTT
CTTCAGTGAATCATCAGAACACGAGTGACGGAAACAAAGGATGTGACAGAGCGAAGTTGGCTTTC
AATTGTTTCACAGAAAATGCTGACAGATATGGATTCGATTTTGACTTTTGA
>SfruOBP12
ATGGGATCGTGCAACGTCTTTGTCGCGCTCGTGGTGTTAACAGTGGGTGTGAGAGAGATAGAACC
ATCCAAGGATGCTATGAAGTACATCACCTCAGGATTCGTGAAAGTTCTGGAAGAATGCAAGCAGG
AGCTAAACATGAACGATCACATCATAGCAGACCTGTTCCACTACTGGAAGCTGGAGTACACGCTG
TTGAACCGCGACACCGGCTGCGCCATCATCTGCATGAGCAAGAAGCTGGAGCTGCTGGACGCCAA
CGGGAGGATGCACCACGGCAATGCGCAGGAGTTCGCCAAGAAACACGGCGCTGGTGACGAAGTAG
CTTCGAAGATAGTGCAAATAATCCACGAATGTGAAAAGAAGCACGAGCGTGATGATGACGAGTGT
CTCCGGGTGCTGGAGGTGGCCAAGTGCTTCCGCACCGGCATCCACGAGCTGGACTGGCAGCCCAA
GGTCGAGGTCATTGTGGAGGAGGTCCTCACGGAAATGTAG
>SfruOBP13
ATGCTTGGTATGAAGACAATCGTAGTGCTCTGTCTGATTGTGTACGAAGCGGGGCTGGTGCACGG
CGAGCACGAGGTGAGCGTGCTGCGCGCCCTCAGCACCAGCATCGGAGACACGCTGCTGGAGTGCC
AGACCGAGCTAGATATCAAGAATGAAGTAATTCAAAGCTTTCTGAACTTCTGGGACCTAAAGAAC
CCCGCGGACACCAAGGAATGGGGCTGTGCTCTTGACTGCGTCTTTCAAAAGAATGCGTTTTTGAC
TCCAGATGGCAAAACCGTGATATCGGCAAACGTGCGAGAATTCTGTAAGGCAGCAGGAGCCGATG
AACTAATGTCGGTGCGGATGGTAGACCTGTTTGAAATGTGCAAGGACGGTGCGAAAAAGATCATA
AGTAAATGTGACAACGCCCTGGAGGTGACCAAGTGTTTCCGCCACGGCATAGTCCAGCTAGATTG
GGCCCCGGAGCACAATTTCTGGAACCGCACTCGGGAGCACCCTGACAACATCCCGGTGCAAAGGA
CAGACATAGTTAAGATTCCTTTGCCGCAGAGACATTCATCATTGAATGTGTGGCGGAGACGATTC
GGTTACAGACGGCTCGCCTCTATCTTAAGAAGATCTTTTTGTTGA
>SfruOBP14
ATGATGAGAAAAAACGTGCCAACCAAAGAATAATGTCGAAGACGAGAAAATCGATCCGATTGCCGA
CGGTGTCTTCATTGACGAGAAAGAAGTAAAATGCTACATGGCTTGTATTATGAAAATGGCTAACA
CGATCAAAAATGGGAAATTAAATTATGATGCAGCCATAAAACAGGCTGATTTGTTACTTCCTGAT
GATATAAAGGAACCTGCTAAGGAAGCTATTACTGCTTGCAGAAAAGTTGGCATGGACTGTAACAT
TGAACACCCTATAACACCAAAAGAAATGCTGGAACTAAAGGATAACAAAATACCTGATACAAACA
ACGCTAAATGTTTCGTTGCCTGCGTTTTCAAGAAAACTGGTATGCTGGACAGCAAAGGCATGTAT
GATGCAGAAAACTCAATAGCAATGACACAAAAAGATTTCGCTAATGACCCGAAGAGATTAGAAGG
TTCCAAGAAACTTTTGGAAACTTGCAAGAAAGTGAATGACGAAGCCGTATCTGATGGTGAAAAGG
GATGTGAAAGATCAGTCCTATTACATAAATGTTTTGTTGAAACCGCGCCTCAG
>SfruOBP15
ATGCTGCTGTTGCTGCGCGCGCTGCCGCTGCTGGCCGCCGTGCTGCCGCTGCGCGCCGACGTCAA

CGTCATGAAGGACGTCACTCTCGGCTTCGGACAGGCGCTCGACAAGTGTCGCCAGGAGAGTCAAC
TGACGGAGGAGAAGATGGAAGAATTCTTCCACTTCTGGCGTGATGACTTCAAGTTCGAGCACCGC
GAGCTGGGCTGCGCCATCCAGTGCATGAGCCGGCACTTCAACCTGCTCACCGACTCCAGCCGCAT
GCACCACGAGAACACCGAGCAGTTCATCCAGTCCTTCCCCAACGGCGAGGTGCTGGCGCGGCACA
TGGTGGAGCTGATCCACGCGTGCGAGAAGCAGCACGACCACGAGGAGGACCACTGCTGGCGCATC
CTGCACGTGGCGGAGTGCTTCAAGCAGGCGTGCGTGCAGCGCGGCGTCGCGCCCTCCATGGAGAT
GATGATGACCGAGTTCATCATGGAGGCGGAGGCGCGCTGA
>SfruOBP16
ATGGAAAAAATACTCATCTTCACGACTTTCCTATCGCTTGCTGTTGTAGTGAACAGCTTCAAAAC
AAAGTTTTTCTCGCAAAGCTTGGACTCGGAACCTCCGGTCTCGATCCTGTACGCGCGTGACAAGA
AAAGTGATAAAGTAACAGATGATTGCTTAACAGAAATGTACCCACGGAACTTGTATAAGTATCCT
CTGCGTATCGATAGGAATGATATACCCTGTATAATACATTGTGTGCTCAAAAAATTCGGTATAAT
GACGAACGATGGTTACATAAACATAAAGAATTACTACAGGAGAGTGCAGGCTATACACAGGTATG
ATCCGAGGATCTTGATATCGGATGTCGGAGAAACCTGTGCTCAGAACATCAACGGAATGAATCTG
GACCACGATGTGTGTAAGAAAGCGAAGGTCTTTAACGATTGCACGCAACTGTACGCAGTGTCCTA
CAAAGACCCCGACGAATGGAAATAG
>SfruOBP17
ATGGCTCAGCATTGTAATAATGCATCTCACGCAGATGTTGTGCAGACTCCTACTAAAAGAGAAAC
TAGAGCTTCTCTGAAACCACTATCAGTATGCTGCGATATCCCAGAGTTGGCTGATGAGAGGCAGT
TATCGAAATGTTCCCCAAAACCTCCTGGCCCGTGTGAAGATGTCCAATGCATCTTTGAAGCATCA
GGCTTCTTGACAGACAAGAATACCTTGAATAAGGCTGCATACAGAAGCCATTTGCAGAAATGGGA
GAAGAGCAATCCGGGCTGGACTGATGCCATTTATAAAGCCATTACTGATTGTGTCGACAATGACC
CAAGGCAGCATCTTGATGTAGCTTGCAAAGCCTATGATGTGTTTACTTGCACTGGTATCGCTATG
TTAAAGAAATGTCCTGAAACAGCGTGGAAGTGTTAG
>SfruOBP18
ATGTTTAAGTTCTGTGTCTTCCTTGCTTTGGGGTTCGTGGCATGCCACGGGGCTCCAAATACTAA
CCCTGGAACTTACTGTGGCGCTACACCAGATAACATGTTCAGGTGCCTGAACAATCCTCGCGTGG
TGACTCCTGAAGTGGCCGCCAAATGTGGCAGTCAAGTCACAGAGTGTGAGAGAATGACCTGCATC
TTCCGTGAATTGAAATGGTCGAAAAAAGGAGCAATAGACAAGGCGAAGGTGAGGGCCTACTTCGA
GCAGTACGAGACGGAGCACCCTGACTGGGCCCAGGCTGTCCAGCATGTGAAGTCCTTCTGCCTGG
TCCCTGAACTTCGGGCCCAGGGAGTGTTCCTCAACTGCCCCGCTTACGACATCATGCAGTGTATC
CTGTCTAGCTTTATCAAGCACGCCAGCCCGTCCGTGTGGTCGAACGCGACAGACTGTGATTACCC
CAAGGCGTTCGCCGCTGCCTGCCCGGTCTGCCCATCTGACTGCTACTCACCCCAGATCCCATTCG
GCTCCTGCAACGCCTGCTACTCACAACCACGTACAGTCTAA
>SfruOBP19
ATGATAACATCATCCAGTTTATTAGTACTAGCTGCTGTTGTTCAGGTGCTATTCGCTCAAGAGCC
TGCCTTCGAGTCTGGACCTCCAGAGCCATGGGGCCCTCCTGAGAAACAGACACCACCCCGCCTTG
CACCAAGAATACCAAGAAACTGTTGGGCCCCTCCACAACGGATAAATGTGTACAAATGCTGCCCC
ATACCTTCACTGTATCCAGATGAAGCCATGCAGAGCTGTGGTTTTGAAAAACTGCCCGAGGGTGA
GGCACCGAAAAAAGCTGTCTATCGTCCCGAGGGTACATGCAAGGAAGGATATTGTGTAATGGAAA
AGTTCAATCTACTTTTGGCAAACAAAAGCGTAGATTACGAAAAATTCGGAAACTATTTAGATAGT
TGGGCGGAGGCGAACCCGGAGTTTGCTAAACCTATTAAACTAGCTAAGGAAGAATGTGCCAAAGA
TGAGGGACCCAAAGGCCCTCCAATCTGCGACCCTGACAGGATATTCTTGTGTCTGACCTCAATAA
TATTTTGGAATTGCGATCTTCGTGATGGAGAAGGTTGCAGTGCATTGCAAGAACACATGAATGAA

TGCCGACAGTACTATACACGC
>SfruOBP20
ATGTTTCGCCGAGCGCTGCTGTTTCTCTCTGTTATTTATTCATTGCAGTATTGCAATGGACAGAC
AGAAGCTCCCGAGAAGAACCGTATGATGGGGATTGACGCTGTTCACGACAACAACGTGAAAATCG
ACAAAGATACTATAATTACACGTAATTTGAAATTGGAGAAACGGAACCGCGGACCGAAGTCGGAT
TCTAATAAAAATGTGGATGAAAAAGAGCCAGACTGGTCTTATGCGAGTTTCCCTAAAGAAATTTC
GGAACACGTTGAGAACTTTAAAAAGAACATGAGCGAGTGTTTGAAGGAAGTGCAGTCTAGTGACA
AGAGGCCTGTCAAGCGTCTCTCTCCTAAAATGGAGTCTCCTGTTCATGGCGAATGTCTCATTGCC
TGTGTTCTGAAACGCAACGGTGTTATTATTCATGGAAAAGTTAATAAAGATAACTTAATAGCTCT
TGTAAGTAAGTTCTACTCTAAAGACACCAGGCTCATGAAGAAATTGGAGAAGAATTTGGATCGTT
GTATCGAAATGAGTGTGCGTGCCCAAGACGACTGCACGTTGGCATCGCTGCTTAACGATTGCACT
AACGACCTAATGGCCAGCAATAAACACAAATTAACGGTTAATTATTAA
>SfruOBP21
ATGTTCAAGTTATCGGTATTTGTGTGTTGTTTGTACTTCTGTGCTTTAACACCTTATTTAGTTTC
TGCCATGACAGCTGAACAGAAGGCTATAATCCATGAACACTTCGAGACCATCGGCAAGGCTTGTA
ACAAAGACAGCACTGTGATAACAGCAGATGATGTCGTCAATCTGCGAGCTAGGAAGATGCCGAGC
GGACCCAATGCGCCTTGCTTCCTGGCTTGCATGATGAAGCAAATTGGCATTATGGATGATAACGG
TATGGTGCAGAAAGAGAATGTGCTTGAAATGGCCAAAGCTGTATTCGACGATCCCGAAGAACTGA
AGGCAATCGAAGACTACCTACACTCCTGCTCACAAGTTAACACTGAGTCGGTCAGTGATGGTGCT
GCCGGCTGTGAGCGTGCTATGCTGGCGTACAAATGCATGATAGAGAATGCTTCCAAGTTTGGCTT
CGATATTTAA
>SfruOBP22
ATGACTTGCTCCCAGGCGCTGGCGCTGCTCGCGCTCGTTGCCATCTCGCAACAGGCAACAACAGG
ATGCAAAAATTGTATAATGCTGGGTAAGGAGGAGAAGGCGATGTTCCGCGCGCACTCGGACGCGT
GCGTGGCGGCGTCGCGCGTGGAGCCGCGCCTCGTGGACGCCATGCTGGCGGGCGAGCTGCTGGAC
GAGCCGGCGCTGCGCAGGCACGTCTACTGCGTGCTGCTCAAGTGCAAGCTCATCAGCAAGGACGG
CAAGCTGCAGAAGGCCGCCGTGCTCGGCAAGATGGCGGCACGACCTGACGCCAAAAACGCTACCA
AGGTTCTAGAGAGCTGTGCAGACCAGACGGGCGACACTCCTGAAGACCTGGCGTGGAACCTGTTC
CGCTGTGGCTACGACAAGAAGGCGCTGCTGTTCGACTACATGCCCACTAACGTCGCCAGCGAGAC
TGACAACAACTCCTAA
>SfruOBP23
ATGATTTTAAGTGTTTGTTACGGTGCTGTTGATATTAAAAAATACTTCAAAGTATGCGACAGAAA
TGCTATAGACGTCAACGACTGCATGGCGGAGGCTGTGCGACAAGGCATTGCAACTATGATCAATG
GGATAGACGAGCTGGGAGTGCCACCGATCGATCCTTACTTACAGAAGGAATTCCGCATGGAGTAT
AAGAATAATCAGATCGCAGCGAAATTGAATTTAAAGAACATTTACGTGGAAGGTTTGAAGGAGGC
CATTGTTCATGACGCCAGATTACGCGCTGATGATGACAAATTCCATTTAGAAGTGGACCTGAGTG
GACCAAGAGTTTCAGTCAAAGCCGATTATTATGGAGAAGGGCAGTTCAACTCTTTGAAGATTGTA
GCCTACGGACAAGTTAATACTACGATGACTGACTTGGTGTACACGTGGAAACTCGACGGAGTTCC
GGAGAAGAATGGCACTGAGACGTATATACGTATCACAGATTTCTACATGAGACCCGACCTTGGCA
GCATTGTCACACTCTTCAAAAACGACAATCCTGAATCTAGAGAATTAACTGACTTGGGTACAAGA
TTCGCCAACGAAAACTGGAGAACGCTTTACAAGGAATTCCTGCCTTATGCTCAAGCTAACTGGAA
GAGAATCGGCGTCAGAGTAGCCAATAAGTTGTTCTTAAAAGTTCCTTACGATCAACTGTTTCCCA
CATCTTCATGA
>SfruOBP24

GAGTCAAGATGCCGCAACCCACCTACAGCGCCACAGAAGATAGAGCGAGTGATCACTCTGTGTCA
GGATGAAATAAAACTGTCGATACTCAGAGAGGCTTTGGACGTGATCAAAGAAGAACACACGATGC
CTGCACAGAGACGGCGTGACAAAAGAGAGGTGCCTTTCACGCACGATGAGAAGAGAATTGCTGGG
TGTCTGCTGCAGTGTGTGTACCGGAAAGTGAAAGCTGTGGACGGCTACGGTTTCCCGACCCTGGA
AGGTCTGGTGGGCCTGTACTCCGACGGTGTGAACGAGCGCGGCTACTTCATGGCGGTCCTGGAAG
CCTCGCGAGAGTGCCTCATGAAGAACCACGACAAATTCTCCAGGACTGTTCCTATGGACAACGGC
CGCAACTGCGATGTCTCCTTCGACATATTCGAGTGTATCTCGGACCGTATCGGCGAATACTGCGG
AACCTCCGGCCTCTAA

>SfruOBP25

ATGGCTAAAGTTACTTGCATACTCCTCTTCGTAGTAGCTGTGAGCTTAAGTAGTGTTCAAACAGA
TGAAAGCCAAAACAGCTCTGAGGTTGATGCAGATGTGGATAAGGTCATAGACGAATGCGCTAAGG
AGTTTCATGTACCACATCAACTGCTCATGGCAGCTGTGACGACGGGCAGTGTTCATGCACTCACC
CCATGTTTTTGGAGTTGCTGTTTCAAGGGAGTTGGAGTCCTGAATAGTGAAGGTCAATACGACAT
CGATGCTACATTGGACTTGTCCAAGAAAATGTTTAGTGGTTCGGAATACCAAAAAGTTGAAGGAA
TCGTAAAATCTTGTGGATCAGTGAACGATATATCAGTAAGCGATGGAAAAGCTGGATGTGAAATT
AGTGTTTCACTAGCAGTTTGCATACTTGATAATTGCAAGAAAATATTCCCAAACATGTTTCATGA
TTAG

>SfruOBP26

GGGTGCGAAAGAGCTGTGCTGGGAGCCATCTGCTACATGGAACATAAAACTGTTCAGGCAGCTGT
TTCTGAAGAAGAGAAGGAAGCCTTCCGTGAGGCGATGGCTCCCATCATTGCGGAGTGTTCCGAGG
AACATGGAGTCAGTGAGGCTGACATCAAAGCTGCGAAGGAGGCAGCCAGTGCTGATGGCATCAAA
CCCTGCTTCTTAGGCTGTGTGATGAAGAAAATTGAAGTGCTGGACTCCAAGGGCTTGTACGACGC
AGAGACTGGACTTGGTAAACTGAAAAAGTTCGTCAAGGATGAAGCTGACTTCGCTAAATTCGAAG
AAATCGCCAAGAAATGCCTTAAAGTTAATGAGGAATCTGTAAGCGACGGTGAAGAGGGTTGTGAA
AGAGCCAAGCTTATTCTGGGTTGCTTCATCGACCACAAAGTTGAAATGCCGTTC

>SfruOBP27

ATGAAGTATAAAACAAGACGCACGCGAAACGAACGCGAGCAAGAAAAAATGCATAAACTACAGAA
CATATTTGCATTAATCTTGTTCATACTATTCTCTTTCGCGTTCTATTTAACTATATCCTTCACGC
CACTTACAAAGGAAGAGCAAATGGAACGCTACAACAAGATGACGGAGAATGTAGAACCATTCAGG
AAGAACTTGACTGAATGTGCGCGACAGGTCAAAGCTTCAATGGCAGACGTCGAGAACTTCATAAA
ACGGATCCCTCAGGCCTCCTTGCAAGGGAAATGCTTCGTCGCCTGCATTCTCAAACGTAACTCTA
TCATAAAGAACAATAAAATAAGCAAAGAACATTTATTAGAAGCCAATAAAGCTGTTTACGGGGAA
GATAGTGAAGTGATGGCTCGTTTAAAGACTGCGGTAGGTGAGTGCACGCAGGTAGTCGAAGGGAT
ATTTGAAGTTTGTGAATATGCGTCGGTTTTCAACGATTGTATGCATATCAAAATGGAGCATATTC
TTGATAAGGTGACGATGGAGCGGCGTATGGAAGCGATAGGCAAGATGACGTCGAACCCTGACCAA
TGGAACGAAGAAGAAGATGAACTCTTGAAGTTGGTTAAAGATGAATTATAG

>SfruOBP28

ATGGTAGGTGCCGTGGCGCTCCTCGCGCTCCTACCGGCTTTGGTCGCGGGTGCTGGTGAAGGCAA
CATCAAATTACTAGAAGATGAAGTAGCAGTAGCTTTGAAATCATGCACGTATCCTGACGACATCA
CTAACTCCAAAGAACCGGCGAGCAAGGAACGTCAACGTCGATCTGAGGACTCTTATGATGCTTCT
CCCAGAATAGACGACAATGTGAAAGAAGGGAATCGGTACAGCCACGAGAGACGAATCAGCAACGA
CAGTGGCGACCAACTAATGGTCATCAACGCAACTGATGACGATTACAGTGGCTACGGATCTGGAA
ATATGGGGGAGAAACTGCTGACATCAGTACCACGCCCCGCGTCTCCGAATAACAACATAAACAAG
AATAATACCAGTAGAACCAGACGAAACGAACCGCTTCTAAACCGCCCAGATTCGGATCAGTGTCT

CAGTCAATGTGTGTTCGCCAATTTACAAGTGGTGGACAGCAGAGGAATACCACGAGAAGCAGAAT
TATGGAACAAAGTGCAATCTGCTGTGACGTCACAACAATCCCGGTCTGCACTGCACGATCAGATA
CGAGCTTGTTTTCAGGAGTTGCAATCAGAGGCTGAAGACAATGGTTGCTCTTACTCCAATAAGTT
GGAGCGATGCCTGATGCTACGTTTCGCCGACCGCAAGGTTGACGGTAAAGGAAATGCTAAGAAAT
CTTCTAAGGAACAAACATGA

>SfruOBP29

ATGTGGAATTCATTATTTTTTCTGTTCACCTTTGGTGTTTTCTCTTTAAATGCTGAGGCTTTAAC
TATGGATGATCTGAAACAGAAATACGTGGACAACATACTAGAATGTAGCAAGCAATACCCCGTAG
ACCGCGCTGATGCAGAACAGCTACAGAACAGAATTATGCCTGATAAAGAATCTGTCAGATGTTTG
TTTGCCTGTGTTTACAAATTGGCGGGAATGATGAACGACCAGGGAGAGCTCTCAGTTGAAGGTGT
CAATGCAATATCTCGAAAGTATTTGGCGGAAGATCCAGAAAAGTTAAAGAAGAGTGAACAGTTTA
CCGAAGCTTGCCGAAGTGTAAACGACGCTCCAGTGAACGATGGAACAAGAGGCTGTGATAGGGCA
GCTTTAATATTCCAGTGTACCATAGACAAATCACCTGATGGTCTCACGGAAGAAGAGCTCAAGAT
GGAGTTCACGAAGTTGGTGATGAAGTGTAACAAGGATGGACAGATGGACATGACAGAACTGGTGC
AGTTGCAGAACTACGTGGTCCCGACGAAGCAGTCGGCCAAATGTGTGTTAGCGTGCGCTTACAAG
GCCGCAGAAGTGATGAATGCTAAAGGTGAATACGACATTGACCACGCGTACAAAGTAGCTGAAAT
GATGAAGAATGGAGATGAGAAACGGCTGGTAAATGCAAAGAAAATGGCAGATCTTTGTGTGAAAG
TAAACGAAAACAGTGTCTCTGATGGCGAAAAAGGTTGTGACCGAGCCGCTATGATATTCAAATGC
ACTGTTGAGAATGCACCCAAGTTTGGATTCAAGTTATAA

>SfruOBP30

ATGGTCGACAAAATCCTGATTTTTACATTTATAACACTCTCCGGCTTCGCTCACGCTCGCATGTC
AGTGATGTATGCCCACGATAAGCTAAGTGACATAGTTGCGCAGCAGTGTCTTTCAGAAATGTACC
CTAAGAACAGGCGCATTGAGATCCAGGAGTCGGACGAACCCTGCATCATCTTCTGCGTCCTCAAA
AAGTTTGGCATCATCAGTGCCACTGGTGTGATCAATCTGGAGGTTTATAGAAAACGAGTCCTAAT
CGCTCACCAGCTAGACCAAAACACCTCAGTCATGGACAACGGGGGCTCCTGCATGGAGAACGCAG
AAGCAACTCAACATAAACAAGACGTCTGCAAGAAAGCCAAGGTCTTCAACGACTGCACACATTTA
TACAGAATACTACTTATGTAA

>SfruOBP31

ATGTGGACGCAGGCGCTAGTGCTGACGCTGGGCGCGCTGGCCTCGCTGGCGGCGGCGGCCGTGGA
GATGGACGAGGACATGGCGGAGCTGGCGCGCATGGTGCGCGACAACTGCGCCGGGGAGACCGGCG
TGGACGTGGCGCTGGTCGAGCAGGTCAACGCCGGCGCCGACCTCATGCCGGACGAGAAGCTCAAG
TGCTACATCAAGTGCACGATGGAGACGGCCGGCATGATGGCGGACGGCGAGGTGGACATCGAGGC
CGTGCTGGCGCTGCTGCCGCCGTCGCTGGCGGACCAGCACGCGCCGTCGCTGCGCGCCTGCGGCA
CCCAGCGCGGCGCCGACCACTGCGACACCGCCTTCCGCACGCAGCAGTGCTGGCAGAAGGCTAAC
AAGGCCGACTACTTCCTCATATAA

>SfruOBP32

ATGAAGACTCTGTTGGTTTTCGCTGCCTGCATTCTGCTTGCTCATGCTCTGACTGATGAGCAGAA
AGAGAAACTCAAGAAACACCGCTCCGAATGTCTGACTGAGACCAACGTTGATGAGCAGTTGGTCA
ATAAGCTCAAGGGTGGTGATTACAAGACGGAGAGTGAGCCCCTGAAGAAGTATGCTCTCTGCATG
ATGATGAAGTCAGAGCTGATGACCAAGGACGGCAAGTTCAAGAAGGACGTCGCTCTGGCCAAGGT
TCCTAACCCAGCTGACAAACCGACCGTGGAGAAGCTTATTGACGCCTGCCTGGCCAACAAAGGCA
ACACTCCTCATCAGACAGCCTGGAACTACGTCAAGTGTTACCATGAAAAAGACCCTAAGCATGCG
ATTTTTTTGTAA

>SfruOBP33

ATGTTCAAGTCAGGTGTTGTGTATTGTATTGTTGTTGCTGTGTTCTTCAAGAATGCTTTGGCCAT
AACTGATGAGTTAAAATCTCATATAGAAGCAAAGTTCTTAACAGTTGGTGCAGAATGTATAAAAG
AGCATCCCCTAACAATTGAAGATCTGTCTGCTTTTAAAAACAGAGTTTTCCCCGAAGGTGAAAAT
GCTGGATGCTTCTCCGCTTGTATTTTCAACAAACTGGGATTGTTTGACGACAAGGGTTCTTTATC
CCACTTAACAGCTCTGGAAAACGCCAAGAAAGTATTTGAAGATCAAGGAGAATTAGAAAATATTG
AGAAATTCCTCACCACTTGTGCTAAAGTCAACGACGAGGAAGTATCTGATGGGGAAAAAGGCTGC
GAAAGAGCAAAATTGGCATACAACTGCTTCATCGAAAATTATGAACAACTCGGATTTGATCTGGA
GTTCTAA

Nucleic acid sequences of SfruCSPs

>SfruCSP1
ATGGTACGTCTTAAAGTTAAGATAGGAATACTTCCAGAGGCGTTGAGGACGAAATGTGTCCGCTG
CACAGAGCGACAGAAGAGAACAGCAGTGAAGGTGATCAAGAGGTTGAAGAACGAGTATCCTGACG
AGTGGTCCAAGCTTGCTTCCCGGTGGGACCCCACTGGAGACTTCACCAGATACTTCGAAGAGTTC
CTCGCTAAGGAACACTATAATACTATTCCAGGATCAGGCAGTGCGCTCCCTACATCGTCTCCGTT
AGCACCACCTCGCGTGCCACAATCAGTACCAGCCACCACCCCGACTCCAGGTCCCACGGAGTCCA
CCCCGCCGAGACCAATCGTACTGAACAGATTTGGTGGCGACGGCGAGCTAATGATGGGCAGTCCC
TCGTCCGCGGGCGTTACTCCAAGGCCGATGACCCAGGCCACCACGAGGCCCAGCACCACCACGAA
GCCTAGTACCACCACGAAGTCGCCTAGCACTAGGCCTGTACCACCCAGACCAACAATGATGACAT
GGGCTGGTGCAGCTTCAAACACGCAGTCGACGCGTTTCCCACTGCGGCCAGTATCAGAAATGTCC
CCACCATACTCCACGGCCATAACCTTCATCGACCAAATCGGTTACAAAATAATAAGGACAACAGA
ACTTGTCACCGACATAGTCAGGAACACAGTGAGAGCTGTCGTTGGTCGGTAG
>SfruCSP2
ATGCAGATCATCTTAGTTTTAATCGTGGCGTGTGTGGGTTTGGCAGCCGGCTTGCATGTGCAGGC
TGGGCCACAAATGACAGACGCACAATTAGAACAGACTTTAGCCGATAAGAGCACGATGCAGCGAC
ACATTAAATGCGCACTCGGTGAGGGACCTTGCGATCCTGTTGGGAGACGCTTACGAACTCTAGCT
CCGTTGGTACTACGAGGGGCTTGTCCACAATGCTCGATGCAGGAGACGCGCCAGATTCGACGCAC
CCTTGCCTTTGTGCAACGCAACTACCCATGGGAGTGGGCGAAAATTGTTCGCCAATATGGTTAA
>SfruCSP3
ATGAAGTCCATGCTTGTGCTCTGCGTGTTGTCTGTGGCGGCGCTGGTGGTGGCGCGCCCCGAGGA
CCACTACACCGACCGCTACGACAACGTGAACCTGGACGAGATCCTCTCCAACCGTCGCCTGCTGG
TGCCCTACGTCAAGTGCACCCTGGACCAGGGCAAGTGCGCCCCCGACGCCAAGGAACTCAAGGAA
CACATCAGGGAAGCTCTGGAGAACGAGTGTGGCAAATGTACCGAGACCCAGAAGAAGGGAACCCG
GCGTGTGATCGAGTACCTGATCAACAACGAGGAGGCGTACTGGAACGAGCTGACGGCCAAGTACG
ACCCTGAGAGGAAGTACACCGTCAAGTACGAGAGTGAACTGAAGAAGATCAAGGCTTAA
>SfruCSP4
ATGAAGTGCATCTACGTGTTGTCTGCTCTGCTGGTCTTTGCTGCTGTCCAGGCTGAGGACAAGTA
CAGCACGGAGAATGATGACCTGGACATCGATGCAGTGGTGGCTGACGTGGATGCTCTCAGGGGAT
TTGTAGGATGCTTCATGGACTCTGTGACCTGCCACGCTGTTGCTGCTGATTTCAAAAAGGATCTC
CCCGAGGCGGTGGCGACGAGCTGCTCTAAATGTACAGAAGCCCAGAAGCATATCTTCCACAGGTT
CCTTCTTGGACTGAAACAGAAGCTGCCTGCTGACTACGAAGCCTTCAAGAAGAAGTTCGACCCTG
AGGGTCAACACTTCCACGCTCTCGAGGCCAATGTCTCCAACTCTTAA
>SfruCSP5
ATGAACGGTGCAAATAAATACAACTTTGGCTTATTGTTAATCTTTATGTGTTTATTTATTGCAAC

TGTTGTTTCTCAAGAGAAATTCTATGATAGAAGATACGATTATTACGAAATAGATACTTTGATAC
AGAATCCTAGATTATTGAAGAAGTATTTGGATTGTTTTCTCTGGAGGGGACCCTGTACGCCTATA
GGCAGAGTGTTTAGACAAATCCTGCCCGAAGCAGTTCAGACGGCGTGTAAGAAGTGCACCCAATC
ACAGCGGAGACTTGCAAGAAAAACATTCGACGCCTTCAAGGGATACTTCCCAGAGATACATGAGG
AACTGAGGAAGAGACTTGACCCCAAAAACAAGTACTATGAAGCCTTTGAGAAGGCTATATCGAGC
GCTTGA

>SfruCSP6

ATGAAGGTACTAGTAGTGTTGTCCGTATTCCTGGCACTTGCAGCTGCAGCGCCACCCAAACCCAT
CACGAAGGAAGAGTTGGCTATACTGGAGGCCTTCGACTACGACGCCGTATTCGCTAATGAAGAGA
GTAGGAAACTAATATTCGATTGCTTGCTAGACAAAGGAGACTGTGGCCCTTACAAAAAAGTTGTT
GAGCTATCAAAGAAATCAATGTATGATGAGTGTGCACTATGTTCCCCAACCCAGATGGCGAAGTA
TGACCGCGTACTAAAACTATTGCACGATGACTATGAAACCTTCTACAATGAATTGATCAAGAAAG
TAGCTTCCGAGAAGGAGTAA

>SfruCSP7

ATGAAATTCGTACTAGTATTGTGCCTGGTGGCCGCCGCAGTCCTGGCTGATGATGACAAGTACAC
CAGTAAATACGACAACATTGATCTGGACGAGATTCTCTCCAACAAGAGGCTCCTCACTGCCTACG
TCAACTGCGTCATGGAGAGAGGAAAGTGCAGCCCTGAGGGCAAGGAACTGAAAGAACATCTAGTA
GACGCCATTGAGACTGGCTGCAAGAAATGTACTGAAGCCCAAGAAAAGGGAGCATACAAGGTCAT
TGAGCACCTGATCCAACACGAGCTGGACACTTGGCGTGAGCTGTGCGACAAGTACGACGCCTCTG
GCAAGTGGAGGAAGACCTACGAAGACCGCGCCAAGGCCAACGGCATCGTCATCCCAGAATAA

>SfruCSP8

ATGAAGGTAGCACTACTCACACTCTGCTTCGCTCTTGGAGTTCTCGCTCAGGATATGTACGAGAA
TGCCAACGACAACTTCGACATCTCTGAGGTTCTGGGCAATGAGAGGCTTCTCAACTCTTATGCCA
AGTGCCTGCTCAACAAGGGACCTTGTACTCCAGAAGTAAAGCAAGTTAAAGATAAATTGCCGGAA
GCCCTGGAAACCCGGTGTGCTAAATGTACAGACAAACAAAAGCAAATGGGTAAGACTTTGGCTCA
AGAAGTTAAGAAAAACCACCCCGACATCTGGAAGCAACTGGTAGAGATGTACGACCCTGAAGGCA
AATACCAACAAGCATGGAAGGACTTCCTCCAAGAATAA

>SfruCSP9

ATGAATTTCCTCGTGTTGTCGATTGTGGTTACGATGGCGGCCTTCGTAGCTGCTGAAACTTACAC
GGACAGATACGACCACATCAACATTGACGAGATCATTGAGAACAGGAAACTGCTGGTGCCTTACA
TCAAGTGCACCCTGGACCAGGGGAGATGTACTCCTGAAGGCAGGGAACTGAAAGCTCACATCAAG
GACGCTATGCAGACCTCCTGCTCGAAATGTACCGAGAAACAAAAGAAGGGTGCCAGGAAGGTCGT
CAGACACATCAGGGCTAAGGAGCAGGATTACTGGAAGCAAATCCTTGCCAAATACGACCCTGAAG
ACCAATACAAGGAGAACTACGAATCCTTCCTCGCCGCTGAGGACTAA

>SfruCSP10

ATGGGTCCACAACCATCTAAACCCGGCATTGCGACAGTAGACGCCGCAAGAGGAGCACTTGCACA
ACTTTCTTTCGGTAAGTATTTTTCTATAAAAAGCTTCTTAGAAAGTGCTGTGAAAGTATTCATAA
ACATGAGGAGCTGGTTGCTATGTCTGTGTGTGCTGACGGTGGTGGTGTCATGCTACTCCCAAGCC
AATCGCTACGAGAACTTCAACCCTGATGCCATCGTGCAAAATGACAGGATCCTGTTAGCTTACTA
CAAGTGCGTGATGGACAAAGGGCCCTGCACGAGAGATGGCAAGAACTTCAAACGTGTCCTGCCAG
AGACGCTAGCCACCGCATGCGGACGCTGCAACCCTAAACAGAAGACGATCGTGCGCAAGCTTCTA
CTGGGCATCAGGTCCAAGAGCGAACCCCGTTTCCTGGAACTTCTAGACAAGTACAACCCTGACCG
CTCCAACAGAGATGCTCTATACGCCTTCCTTGTCACCGGTGCTTAA

>SfruCSP11

ATGAGGGTCCTGGTTGCTTTGTCGTGCTTGGTCGTGGTGGCGTTGGCTGCTGATAAATACAACGC
TAAATATGATAACTTTGATGTGGATACCCTGATCTCTAACGACAGACTGCTGAAGGCATACATCA
ACTGTTTCTTGGATAAGGGACGGTGCACGCCCGAAGGATCAGATTTCAAAAAGGCCCTCCCTGAG
GCGATAGAGACAACTTGCGCCAAGTGCACAGAAAAGCAGAAGGGTAACATCAGGAAGGTGATCAA
GGCCATCCAGCAGAAACACCCCAAGGAGTGGGAAGACCTCGTTAAGAAGAACGACCCCAGTGAGA
AGCACCGCGCTAACTTCGACAAATTCCTTCAGGGAAGCAGCTAA
>SfruCSP12
ATGAAACTGGTAATCATTTTGGCATTGGTGGCGGTGGCTGTAGCCCGCCCTGATGACGGTGGCAC
CTATGACTCGAAGTACGACAACTTCAATGTTGATGAGCTCGTTGGAAACGCACGGTTGTTGAAAT
CGTACGCCCACTGTTTCCTTGATGACGGCAAGTGCACTCCCGAAGGAAACGATTTTAAGAAATGG
ATCCCTGAAGCTACGACCACATCTTGTGGAAAGTGTTCAGAGAAGCAAAAGGCTCTCGTTGCAAA
AACCATCCAAGCTATCAAGGACAAACTTCCAGCAGAATATGAAGCTCTAATAAAGAAGCATGACC
CTGAAAATAAACACCAAGGCAAACTCCAGGAGTTCCTTCAAAAATACTCTCATTAA
>SfruCSP13
ATGAAGAGTATCCTAGTTTTGTGCTTGCTGGTCGCGGCCGTCTCCTGCAAACCAGAGACTTACGA
CACTAGATATGACAACTTCGACGTTGAATCCCTGGTCGGCAATGTTCGGCTGCTGACTGCGTACG
GCCACTGCTTCCTTGGCAACGGACCCTGTACTCCCGAGGGCAGTGACTTCAAAAAAACCATCCCC
GATGCTCTGAGGACCGGCTGTGGCAAGTGCTCGCCGAAACAGCGTCACTTGATCCGTGTGGTTGT
AGCAGGCTTCCAGAGCAAGACCCCAGACCTTTGGCAGCAGCTGGTCAAGAAGGAGGACCCCAACG
GACAGTACAAAGAAATCTTCACCAGATTCCTGAACGCCAGAGACTAA
>SfruCSP14
ATGAAGGTCGTGTTTCTCGTGTGTGTGTTGGCTGCGGTGGTGTACGGTGAGCAGTATACGGACAA
ATATGACAACATCGACCTGGACGAGATCCTTCATAACGAAAAAATCCTGCAATCTTATGTAAACT
GCTGCCTGGACCAGGGCAAGTGTACACCTGACGGCAAGGAATTGAAATCGCACATTAAGGAAGCT
TTAGAAAACCGATGTGCAAAATGTACTGATGCTCAGAAGAGAGGTACAGAGAAGGTCCTGAAGTT
CCTCATCAACAATAAGCCAGACATCTGGGACCAGCTCTGCAACAAATTCGACCCCGAAGGATTAT
ACAGGAAGAAGTACGAACAGGAGTACCAATCCGCTAAACAATAA
>SfruCSP15
ATGCAGTATATATTCCGTTATGGGCGAGATGAAGGCGTCACAGCCCAGCGAAGGGTCGAACGGAG
CTCACGGGAAATACGATACAAAATGAAACTCATCATCGTACTCGCTTTATGTGTGGTGGGAGTCG
CCTGGGCTAGGCCTGCCTCCACTTACACTGACAAGTGGGACAACATCAACGTAGACGAGATCTTA
GAGTCCCAGCGTCTACTGAAAGGGTACGTAGACTGCTTGCTCGAAAGAGGACGTTGCACTCCTGA
TGGCAAAGCCCTCAAAGAAACTCTTCCTGACGCCTTAGAGCATGAGTGCAGCAAATGTACGGAAA
AACAAAAGAAGAGTTCAGACAAGGTCATTAGGTTCTTGGTGAATAAACGCCCCGACTTATGGCAG
GAGTTGTCAGGAAAGTACGACCCTGAGAATATCTACCAGGAGAGGTACAAGACTCAGCTCGAAGC
TGCCAAGAAACATTAA
>SfruCSP16
ATGTATGGTATAAAACTGGATGAATGGTGGCTTCAACCATCGATTCCACTGGGACATCGGATCAT
ACACATTGATACAAAATTCAATATGAACGCGCTACTGATCGCTGTTTTCGCCTTAGCCGCTCCTC
TTGCTTATGGCTATGACGAGAAATATGACAAAATAGACGTAGACAAGATTTTGGGGGATGATGCA
CTATTCACCACCTACATTAATTGCATGCTGGACAAGGGACCCTGCTCGGTAGAACATTCTGCTGA
CTTCAGACAACTGCTGCCCGAAGTGATAGCCACAGCGTGCGAGAAATGCAATGCCATACAGAGGC
AAAATGTCAGGAAGACAGTCAAAGCCCTCAGTGAGAAGAAACCTGATGAGTTCGCCCAATTCCGA
GCCAAATTCGACCCTAAGGGCGAATACGAGAAGGCATTCAGTGCTTTCGTCATTGGTACAGATTA

A

>SfruCSP17

ATGAAGGCTGACTGTGTGTTAATTATGACGTTGATGGCAGTGGTCGCAGCTGACTTCTACAACTC
CAAGTATGACAATTTCGACGTGCAGCCCTTGTTGGAAAACGATAGGATACTACTGAGCTACACCA
AATGCTTTCTAGACCAAGGACCCTGTACGCCTGATGCCAAAGATTTTAAAAAAATAATACCAGAG
GCTCTAGAAACTACGTGTGGTAAATGCAGTCCTAAGCAGAAGCAATTAATTAGGACAGTGATCAA
GGCTGTGATAGCTAAACACCCTGAGGCTTGGGAACAACTCAGTGACAAATATGATAAGGACAAAA
AGTATAAAGACTCCTTCGACAAATTCTTAGCCGAACAAGAATAA

>SfruCSP18

ATGGGAAATTACGTGGAAATTATAGTCTTGGAACTTAAAGAAGATAAGTTGCTGACAAAGGCACT
ACACCGTGAAATTATGGGTTTGATGGACGACAGTGAGGTGTGTAATGACACACTAGAACCCCATT
ACATGTATACTGTGTTCACCATGAGAAGTGCAGTAGTTCTGTGGAACATGATTGTTGAGGTTACT
TTGACTGTGGATAGTGCAGTAAGACAGAAAATGAAGTCCTTCATAGTTTTGTGTTTGTTCGGTCT
GGCTGCGGTGGCTCTGGCCAAGCCAAATGGCAGCACTTACACCGACAGGTACGACAATGTCAACC
TGGATGAGATCCTCGGAAACCGTCGTCTCCTGACTCCTTACATTAAGTGCATCCTCGAAGAGGGC
AAGTGCACTGCTGATGGCAAGGAACTGAAATCTCACATCAGGGAAGCCTTAGAACAGAACTGTGC
CAAGTGTACTGATGCTCAGCGCAGTGGAACCCGCAGGGTCTTGGGCCACCTCATCAACAACGAAG
AGGAGTCCTGGAACCGCCTCAAGGCCAAATACGACCCTCAGTCCAAATACACCGTCAAATACGAA
CTCGAACTCAAGAAATTGAAACAATAA

>SfruCSP19

ATGAAGTTCGTATTAGTAGTCTTCGTTGCAGCCTTTGCGGTGGTAATGGCTGAAGAATCGTACGG
AACACAGTATGATAATGTAAATGGAGAAGCTATCGTTTCTGACGATAAACAGTTCCAAAGCATCG
TCGACTGTTTCATGGGCGCTGATTCTTGTAACGAACTTGCTGCTGCATTCAAAAAAGTTCTACCT
GAAGCGATTGTACAAGCTTGTGCTAAATGTACTTCAGCACAGAAGCACCTAGCGAGGGTCTTCAT
AGAAGCATACTCAAAGAAAATGCCACAAGATTATGAAAAGTTCAAAGATTTATTTGATCCTGAAA
GAAAATACTTTGCCAAATTCGAAGCGTCTGTTACCGGATTTTAA

>SfruCSP20

ATGGGTAATTGCTGTTCTAAATTACCTACTATGATCAAATACGCGTTGCTGTTATGTTGTGTGGC
CGCGGTGTCCGTGGCGCAGTCCCAAAGGCCTCCAGTCTCCGACACAGCCCTTGATGATGCCCTAC
AAGACAAACGTTTCATTCAGAGACAGCTAAAGTGCGCGCTGGGGGAAGGACCTTGTGATCCCATC
GGGAAAAGACTTAAAACACTAGCACCCCTGGTACTTCGAGGAGCCTGTCCCCAGTGCACGCCACA
AGAAACCAAGCAAATCCAACGCACTCTATCGTACGTGCAAAGAAATTATCCGCAGCAGTGGGCGA
AAATAGTGCGCCAATACGCAGGATAG

>SfruCSP21

ATGTGGGCTCAGGCGATTCCATACCTGATCGTAGTACATGTGGCGATGGGAGAACAAAAAGTGCC
AGGGTTCTCTAGAGAAATGAGCGACGGAGTAGAAAGCAGAGGCTTTAAGATCATTTACGGCGATG
AAGACATGACCATCATCAACCAAGTGGTCAGTGAAGCCGAAAAAGGCAGTGCTTTTAAGAAGAGA
GTACGTTTGAATGAAGCTATTAAACCCTTACCGGCGCAAGATGTCAAGTGCTTGATGTCTGTCGA
CCGGTACTGTAGCAAGGAGATGGGCATGATGAAAAGTGTATTGATACAAGCTGTAAAAGATGACT
GCGCGAAATGTTCAGTGGAGCAGAAGGATCAAGCTGGGAAAGTAATTGCATCCATGATGGCTCAT
GATCCAGTCGCGTGGAAAGTTTTCCTCACCAGGTACGACGGCATCAAAAAAGTCCAAAGGATACT
AGGATAG

>SfruCSP22

ATGGCTTCCTCATACTATTCAGTGCTCACTGTGAACGAGACTGTTATTTCTTCTGCTGTTCCTGG

AATGATGAAGAACTCGTATAAAACAATGATACTCTTAGTATACCTGACAATTCAGAGCAACGCGA
CAGAGACTTCCACATACACGACCAAATATGATGGCATCGATCTCGACGAAATACTAGACAACGAC
CGTCTACTGACTGGGTATGTCAACTGTCTGATGGATCTTGGTCCTTGCACGGCTGATGGAAAGGA
ATTGAAAAAGAACCTTCCGGATGCTATAGACAATGACTGTAAGAAGTGCACTGAGAGACAGCGTG
AGGGCGCTGACCGTGTCTGTCATTACCTCATCGACAACAGACCTGAAGACTGGACCAAACTTGAG
GAGAAATACAAATCTGATGGAAGCTATAGAGCTAAATATTTAGCCAGTAAGCAGACTAAAGATGA
GAAGGAATCAAATGCCACAAAATCCAGTGAGGATACAAATAATGTATCTAAAGAATAA

Supplemental Table S6. qRT-PCR primers of selected *SfruOBP*s and *SfruCSP*s

Gene	Forward primer (5'→3′)	Reverse primer (5'→3′)
Rpl32	TACAATCGTCAAAAAGAGGACGA	AAACCATTGGGTAGCATGTGA
OBP17	AATGTTCCCCAAAACCTCC	GCTTCTGTATGCAGCCTTAT
OBP22	CTACTGCGTGCTGCTCAAG	TGTCGCCCGTCTGGTCTGC
OBP24	TCACGCACGATGAGAAGAG	CAGGACCGCCATGAAGTAG
OBP32	ACCGCTCCGAATGTCTGACTGA	CGTCCTTCTTGAACTTGCCATCCT
CSP3	TTCCGACAAACCTGTAACCAACCT	GCTTCCCTGATGTGTTCTGTGTGA
CSP19	AGCCCTTGATGATGCCCTAC	CGCACTATTTTCGCCCACT
CSP20	CCTTTGCGGTGGTAATGGC	TCGCTAGGTGCTTCTGTGC
